# Accurate segmentation of intracellular organelle networks using low-level features and topological self-similarity

**DOI:** 10.1093/bioinformatics/btae559

**Published:** 2024-09-20

**Authors:** Jiaxing Huang, Yaoru Luo, Yuanhao Guo, Wenjing Li, Zichen Wang, Guole Liu, Ge Yang

**Affiliations:** State Key Laboratory of Multimodal Artificial Intelligence Systems, Institute of Automation, Chinese Academy of Sciences, Beijing 100190, China; School of Artificial Intelligence, University of Chinese Academy of Sciences, Beijing 100049, China; State Key Laboratory of Multimodal Artificial Intelligence Systems, Institute of Automation, Chinese Academy of Sciences, Beijing 100190, China; School of Artificial Intelligence, University of Chinese Academy of Sciences, Beijing 100049, China; State Key Laboratory of Multimodal Artificial Intelligence Systems, Institute of Automation, Chinese Academy of Sciences, Beijing 100190, China; School of Artificial Intelligence, University of Chinese Academy of Sciences, Beijing 100049, China; State Key Laboratory of Multimodal Artificial Intelligence Systems, Institute of Automation, Chinese Academy of Sciences, Beijing 100190, China; School of Artificial Intelligence, University of Chinese Academy of Sciences, Beijing 100049, China; State Key Laboratory of Multimodal Artificial Intelligence Systems, Institute of Automation, Chinese Academy of Sciences, Beijing 100190, China; School of Artificial Intelligence, University of Chinese Academy of Sciences, Beijing 100049, China; State Key Laboratory of Multimodal Artificial Intelligence Systems, Institute of Automation, Chinese Academy of Sciences, Beijing 100190, China; School of Artificial Intelligence, University of Chinese Academy of Sciences, Beijing 100049, China; State Key Laboratory of Multimodal Artificial Intelligence Systems, Institute of Automation, Chinese Academy of Sciences, Beijing 100190, China; School of Artificial Intelligence, University of Chinese Academy of Sciences, Beijing 100049, China

## Abstract

**Motivation:**

Intracellular organelle networks (IONs) such as the endoplasmic reticulum (ER) network and the mitochondrial (MITO) network serve crucial physiological functions. The morphology of these networks plays a critical role in mediating their functions. Accurate image segmentation is required for analyzing the morphology and topology of these networks for applications such as molecular mechanism analysis and drug target screening. So far, however, progress has been hindered by their structural complexity and density.

**Results:**

In this study, we first establish a rigorous performance baseline for accurate segmentation of these organelle networks from fluorescence microscopy images by optimizing a baseline U-Net model. We then develop the multi-resolution encoder (MRE) and the hierarchical fusion loss ($L_{hf}$) based on two inductive components, namely low-level features and topological self-similarity, to assist the model in better adapting to the task of segmenting IONs. Empowered by MRE and $L_{hf}$, both U-Net and Pyramid Vision Transformer (PVT) outperform competing state-of-the-art models such as U-Net++, HR-Net, nnU-Net, and TransUNet on custom datasets of the ER network and the MITO network, as well as on public datasets of another biological network, the retinal blood vessel network. In addition, integrating MRE and $L_{hf}$ with models such as HR-Net and TransUNet also enhances their segmentation performance. These experimental results confirm the generalization capability and potential of our approach. Furthermore, accurate segmentation of the ER network enables analysis that provides novel insights into its dynamic morphological and topological properties.

**Availability and implementation:**

Code and data are openly accessible at https://github.com/cbmi-group/MRE.

## 1 Introduction

**Figure 1.**
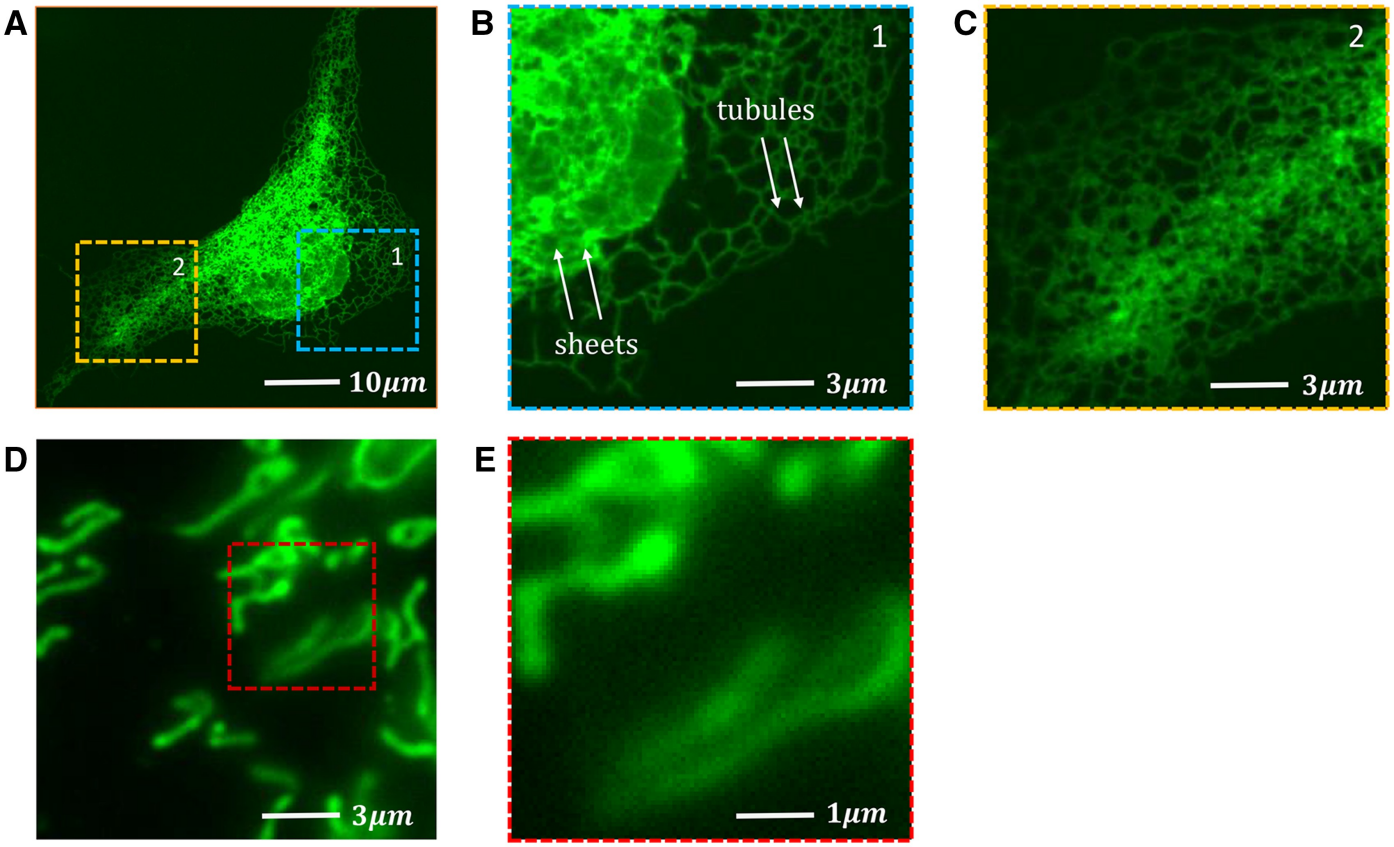
Challenges in segmentation of the ER network and the mitochondrial network. (A) The ER network in a cultured COS-7 cell. (B) Magnified view of the first rectangular region in (A), with arrows pointing to representative ER tubules and sheets. (C) Magnified view of the second rectangular region in (A). The complexity and density of the network pose a substantial challenge for segmentation. (D) A mitochondrial network in a cultured COS-7 cell. (E) Magnified view of the rectangular region in (D). The blurring of boundaries of mitochondria poses another substantial challenge for segmentation.

Intracellular organelle networks (IONs) such as the endoplasmic reticulum (ER) and the mitochondrial (MITO) networks serve crucial functions in cell physiology. Morphology of these networks is critical in mediating their functions (Friedman and Nunnari 2014, Westrate *et al.* 2015). For example, the morphology of the ER network plays a critical role in mediating the metabolic and signaling functions of the network in normal cell physiology and in driving cellular dysfunctions in human metabolic and neurodegenerative diseases (Westrate *et al.* 2015, Lee and Blackstone 2020). Accurate segmentation is essential to characterizing the morphology and topology of IONs for applications such as disease mechanism analysis (Westrate *et al.* 2015, Lee and Blackstone 2020) and drug target screening (Boutros *et al.* 2015). However, the structural complexity and density of these networks pose substantial technical challenges. In particular, the ER network is among the most complex intracellular structures visualized by fluorescence microscopy (Fig. 1A–C).

To overcome the technical challenges posed by the structural complexity and density of organelle networks, we choose deep learning models for their excellent performance in challenging image segmentation tasks (Shen *et al.* 2017, Garcia-Garcia *et al.* 2018, Minaee *et al.* 2021). However, most of these models are designed originally to segment natural images. They are not optimized for biological fluorescence microscopy images, whose distinct properties such as low signal-to-noise ratios, hybrid noise distributions, and blurred object boundaries (Fig. 1A–E) differentiate them from natural images in important ways.

In developing deep learning models for accurate segmentation of fluorescence microscopy images, there is often an emphasis on designing novel architectures. However, recent comparative studies on metric learning (Musgrave *et al.* 2020) and network pruning (Blalock *et al.* 2020) have highlighted the importance of establishing rigorous and consistent baselines when comparing the performance of different architectures or algorithms. Motivated by these studies, we use ablation to optimize the preprocessing methods and the loss functions of our baseline model, the U-Net, a widely adopted deep neural network for the segmentation of biomedical images (Ronneberger *et al.* 2015). This optimization alone substantially enhances the baseline performance, providing a technically rigorous and fair reference for performance comparison with other models.

Because of the structural complexity and density of the organelle networks, their fine structural details cannot be effectively represented by the high-level semantic features obtained after multiple layers of convolution operations. Therefore, it is essential to use low-level features for accurate segmentation, as demonstrated previously in (Zhou *et al.* 2019, Guo *et al.* 2021). In addition to their structural complexity and density, the ER and MITO networks exhibit topological self-similarity. Namely, they exhibit self-repeating patterns at different scales so that these complex networks can be represented by simple structural units (Song *et al.* 2005). This property aids models in understanding complex network structures, thereby improving segmentation performance (Huang *et al.* 2024). To exploit the low-level features and the topological self-similarity of the organelle networks, we introduce the multi-resolution encoder (MRE) and the hierarchical fusion loss ($L_{hf}$). MRE captures low-level features spanning multiple scales by random cropping of the input image, while $L_{hf}$ imposes constraints on topological self-similarity by integrating outputs across layers at multiple scales.

**Figure 2.**
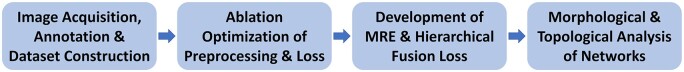
Overall workflow of this study.

Figure 2 summarizes the overall workflow of our study. First, for the training and testing of different segmentation models, two datasets, ER and MITO, are constructed for the ER network and the mitochondrial network, respectively (Luo *et al.* 2020). Next, ablation is used to optimize the preprocessing methods and the loss functions of the baseline U-Net model. Then, MRE and $L_{hf}$ are developed to capture the low-level features and topological self-similarity of IONs to enhance segmentation performance. Lastly, the segmentation results are used for downstream morphological and topological analysis of the organelle networks. In summary, the main research contributions of this study are as follows:

1. We have developed new deep learning model architectures by introducing MRE and $L_{hf}$, which enhance the performance of both convolutional and Transformer-based segmentation models in overcoming the challenges posed by the structural complexity and density of intracellular organelle networks. By leveraging low-level features and enforcing topological self-similarity, MRE and $L_{hf}$ enable models such as U-Net, HR-Net, PVT and TransUNet to outperform competing state-of-the-art models in segmentation performance. Furthermore, these improvements have been validated in the segmentation of the retinal blood vessel network, confirming their generalization capability in segmenting complex network structures.
2. Our study demonstrates how to optimize deep learning models for segmentation of fluorescence microscopy images, whose distinct properties differentiate them from natural images in important ways. In particular, our study highlights the importance of low-level features. Many deep learning models are designed originally for natural images. Without optimization, they may perform poorly on fluorescence microscopy images.
3. We have developed resources for further studies of intracellular organelle networks, a distinct class of dynamic and distributed networks for bioinformatics research. The resources include open-access datasets of intracellular organelle networks, ER and MITO, to support the development of new deep learning segmentation models. We have also developed methods and software to analyze dynamic morphological and topological properties of intracellular organelle networks.

## 2 Related works

### 2.1 Segmentation of fluorescence microscopy images

Both conventional and deep learning methods have been developed for the segmentation of fluorescence microscopy images (Cremers *et al.* 2007, Srinivasa *et al.* 2009, Xing *et al.* 2017, Gupta *et al.* 2019, Moen *et al.* 2019). In conventional methods, image objects are represented by hand-crafted features such as intensity, color, texture, and shape. Representative examples of these algorithms include level-set (Cremers *et al.* 2007), active mask (Srinivasa *et al.* 2009) and graph-cut (Peng *et al.* 2013). Typically, they are customized for certain classes of images or applications. Their performance relies heavily on the effectiveness of hand-crafted features. Consequently, parameter tuning is often required. These algorithms are generally unsupervised but often exhibit unsatisfactory generalization capability and robustness.

Over the past decade, deep learning has reshaped nearly all areas of computer vision. Deep learning models, which learn to represent image objects through training, have achieved breakthrough performance in the segmentation of complex natural and medical images (Shen *et al.* 2017, Garcia-Garcia *et al.* 2018, Minaee *et al.* 2021). They have also achieved remarkable success in the segmentation of biological fluorescence microscopy images (Xing *et al.* 2017, Gupta *et al.* 2019, Moen *et al.* 2019). Currently, architectures of deep learning models need to be customized for specific types of images or applications, as illustrated, e.g. in a recent comparative study on cell nucleus segmentation (Caicedo *et al.* 2019b). Various techniques have also been developed for a comprehensive search of the network architecture space (Talbi 2021). However, the adoption of these techniques has been limited by factors such as their limited configuration space and high computational cost. In practice, customized model architectures need to be integrated with customized selection or design of preprocessing methods and loss functions (Caicedo *et al.* 2019a, b).

### 2.2 Segmentation of the ER and MITO networks

The ER is a continuous network of thin tubules and interspersed sheets that extend throughout the entire intracellular space (Fig. 1A). Because of its critical importance in mediating ER functions, ER morphology has been quantitatively characterized in multiple studies (Pain *et al.* 2019, Lu *et al.* 2020). However, the structural complexity and density of the ER network pose substantial challenges to image segmentation. In several related studies, the ER network is segmented either manually (Pain *et al.* 2019) or using some variants of intensity-based thresholding (Pain *et al.* 2019). The robustness of these methods is generally unsatisfactory because their performance depends strongly on image conditions such as signal-to-noise ratios and levels of blurring. Recently, deep network models have been proposed to segment the ER network (Lu *et al.* 2020, 2023). However, how the proposed models compare in performance with other state-of-the-art deep learning segmentation models remains to be examined.

Mitochondria form a network through their fusion and fission (Friedman and Nunnari 2014). Although they often cluster in the intracellular space, their network generally is not as continuous as the ER network. Individual mitochondria or their clusters exhibit complex and irregular morphology. Furthermore, due to their varied poses in 3D, their boundaries are often blurred (Fig. 1D). A variety of conventional techniques have been proposed for the segmentation of the mitochondrial network, including active mask, top-hat, level-set, and intensity thresholding (Leonard *et al.* 2015, Rohani *et al.* 2020). Recently, a modified U-Net has also been proposed (Fischer *et al.* 2020). However, its performance in comparison with other state-of-the-art deep network segmentation models remains unclear. Overall, reliable and accurate segmentation of the mitochondrial network remains technically challenging.

## 3 Materials and methods

### 3.1 Construction of custom datasets

We have developed two fluorescence microscopy image datasets, named ER and MITO, for organelle network segmentation. Expert-annotated binary masks were produced as ground truth. Further details on these datasets are provided in the Supplementary Material and at IEEE DataPort (DOI: 10.21227/t2he-zn97).

### 3.2 Ablation optimization of preprocessing and loss

We choose U-Net as the baseline model and conduct an extensive ablation study to optimize its preprocessing methods and loss functions. Detailed descriptions of preprocessing methods and the loss functions are provided in the Supplementary Material.

**Figure 3.**
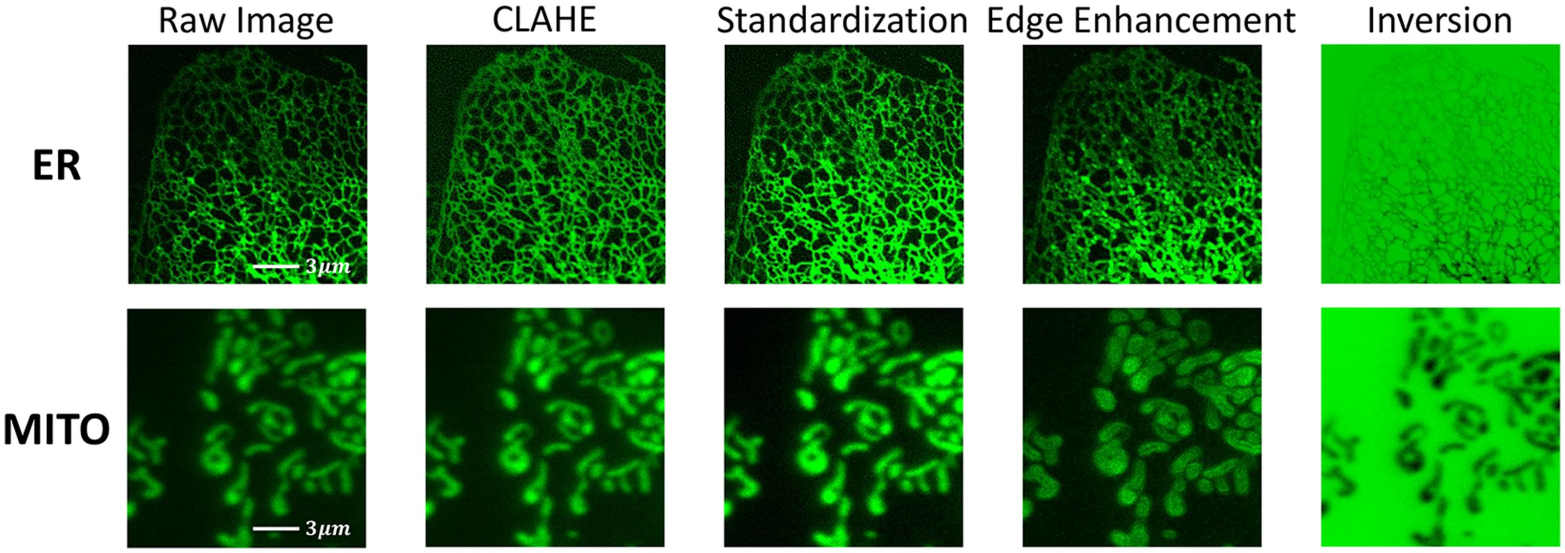
Effects of different preprocessing methods.

**Preprocessing methods.** The methods tested include Equalization (CLAHE), edge enhancement (Edge), standardization (STD) and inversion (Fig. 3). These methods counteract challenges in segmenting fluorescence microscopy images with high bit-depths and dynamic ranges, such as boundary blurring and thin structure visualization.

**Loss functions.** Five distinct loss functions are tested, including binary cross entropy (BCE), weighted binary cross entropy (WBCE), soft intersection over union (Soft IOU), dice coefficient, and focal loss.

**Preprocessing methods and loss functions.** Separate optimization of the preprocessing methods and loss functions narrows the range of our selections. We also test the performance of different combinations. See Section 4 and the Supplementary Material for further details.

### 3.3 Optimization of model architecture

#### 3.3.1 Distinct characteristics of IONs

We highlight two distinct characteristics of the organelle networks that can be used to enhance segmentation performance.

**Low-level features.** Objects in natural images usually contain both low-level and high-level features. For example, a hierarchical chain of low-level to high-level features of a person *may be composed of point, line, circle, eye, face, and person*. Fluorescence microscopy images usually contain only one semantic class in the foreground of each wavelength channel because of molecular specificity of fluorescence labeling. Organelle networks are simple in their semantic structure and mainly consist of low-level features. For example, a hierarchical chain of features of the ER network may be composed of *point (junction), line (tubule), polygon (mesh), and network (ER)*. Overall, a segmentation model for organelle networks should pay more attention to their low-level features.

**Topological self-similarity.** Another characteristic of the organelle networks is their topological self-similarity, which means that large and complex organelle networks have similar topological patterns at different resolutions (scales). For example, in Fig. 1, if we consider “one junction with multiple edges” as a primary structural component of the ER network, different sections of the network at both the global and local scales contain similar topological information, with the main difference being in the number of primary structures. This topological self-similarity can be exploited to extract similar low-level features at different resolutions from the organelle networks.

#### 3.3.2 Development of MRE and $L_{hf}$

Considering the significance of *low-level features* and the *topological self-similarity* inherent within IONs, we develop a multi-resolution encoder (MRE) aimed at capturing low-level features across multiple scales. Furthermore, a hierarchical fusion loss is devised to leverage the self-similar characteristics of images. This dual approach is proposed to enhance performance of models specifically for the segmentation of IONs.

Specifically, for the MRE, we denote the original image as $I_{\mathrm{raw}}\in R^{C\times H\times W}$, where (C,H,W) denotes its color channel number, height, and width, respectively. In this study, we use grayscale images so C=1. For the $j_{th}$ convolution or Transformer stage, j∈{0,1,2,3,4}, we generate five multi-resolution images $I_{j}\in R^{C\times H_{i}\times W_{i}}$ as input, where $H_{j}=\frac{H}{2^{j}}$, $W_{j}=\frac{W}{2^{j}}$. Note that we randomly crop from $I_{\mathrm{raw}}$ rather than use linear interpolation to generate multi-resolution images (Fu *et al.* 2018). This preserves topological information for the multi-layer inputs. The random cropping is performed in the same way in both model training and model inference.

**Figure 4.**
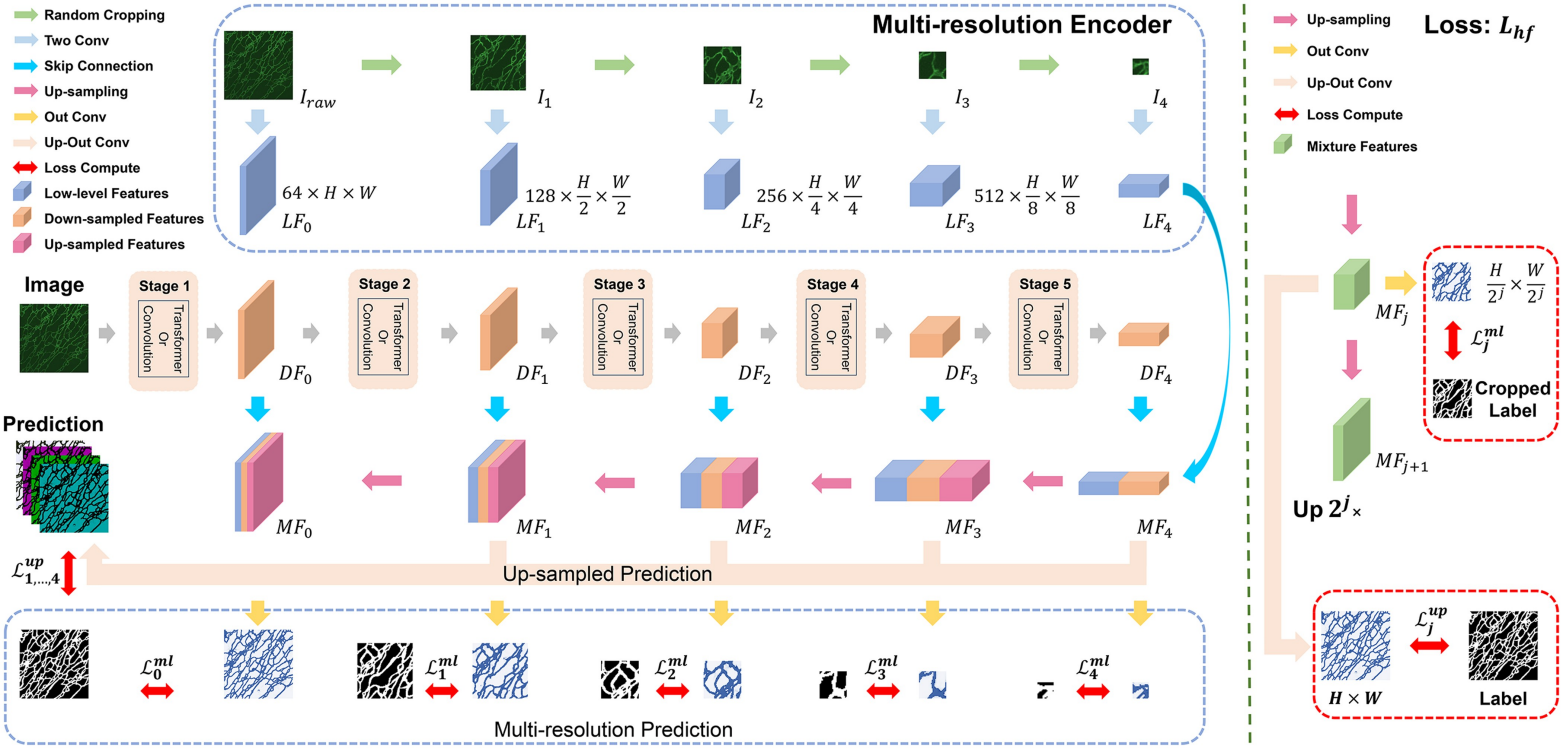
Left: Architecture of the MRE. Right: Workflow of computing up-sampled loss and multi-resolution loss. Each blue rectangular block corresponds to a low-level feature $LF_{j}$. Each orange block corresponds to a down-sampled feature $DF_{j}$. Each red block represents an intermediate feature map generated by up-sampling layer. The mixture feature map $MF_{j}$ (green rectangular block) is a horizontal concatenation of $LF_{j}$ and $DF_{j}$.

To extract low-level features $LF_{j}$ of each input image $I_{j,j\in \{0,1,2,3,4\}}$, we use two 3×3 convolution filters whose channels are doubled from its previous layer (64→128→256→512→1024). The extracted five low-level features $LF_{j,j\in \{0,1,2,3,4\}}$ are then concatenated with the down-sampled features from the normal encoder to generate the mixture feature maps $MF_{j,j\in \{0,1,2,3,4\}}$, which are used for skip connecting from the encoder to the decoder. For each up-sampling layer in the decoder, the up-sampled features are first concatenated with $MF_{i}$, followed by two 3×3 convolution filters to reduce the channels to a half. After four up-sampling layers, we obtain a prediction with the size of H×W (Fig. 4).

To exploit self-similarity within intracellular organelle networks, a 1×1 convolution filter is used on each mixture feature maps $MF_{j,j\in \{0,1,2,3,4\}}$ to output five multi-resolution predictions. As with the input images, the five labels are also cropped from the original masks. To use the label information effectively, each up-out layer also generates a prediction with the same size as the original image by an independent deconvolution layer. In summary, we obtain nine segmentation predictions, five of which are the multi-resolution outputs, and the other four are up-sampled to the same size as the origin image. The overall architecture of MRE and the workflow of computing $L_{hf}$ is shown in Fig. 4.

We compute the hierarchical fusion loss $L_{hf}$ as follows:

(1) $$L_{hf}=L_{up}+L_{ml},$$

(2) $$L_{up}=\sum_{j=1}^{4}\alpha_{j}L_{j}^{up},$$

(3) $$L_{ml}=\sum_{j=0}^{4}\beta_{j}L_{j}^{ml},$$

where $L_{up}$ denotes the four up-sampled losses, and $L_{j}^{up}$ is calculated between the up-sampled predictions of the $MF_{j}$ and the original labels. $L_{ml}$ denotes the five multi-resolution losses, where $L_{j}^{ml}$ is calculated between the $MF_{j}$’ prediction and the corresponding cropped label. $\alpha_{j}$ and $\beta_{j}$ are the weights of $L_{j}^{up}$ and $L_{j}^{ml}$, respectively. $\beta_{0}$ is set as 0.5 to guide the model to pay more attention to the losses from the original resolution, while $\alpha_{j}$ and $\beta_{j}$ are set as 0.0625 when *j* equals to 1, 2, 3, and 4. The sum of $\alpha_{j}$ and $\beta_{j}$ is 1. Based on previous ablation optimization of the loss functions, Soft IOU is chosen for all losses:

(4) $$L_{j}^{up}=L_{j}^{ml}=1-\frac{\sum_{i=1,j=1}^{H_{i}W_{j}}{\hat{y}}_{i,j}y_{i,j}}{\sum_{i=1,j=1}^{H_{i}W_{j}}\left({\hat{y}}_{i,j}+y_{i,j}-y_{i,j}{\hat{y}}_{i,j}\right)+\eta },$$

where $\left(y,\hat{y}\right)$ denote the pixel-level ground-truth and the prediction, respectively.

## 4 Experiments

### 4.1 Experimental setup

**Datasets.** For training, testing, and optimization of deep learning models, we use our custom ER and MITO datasets. To test the generalization capability of models, we use two public datasets of the retinal blood vessel network, namely DRIVE (Staal *et al.* 2004) and STARE (Hoover and Goldbaum 2003). See the Supplementary Material for details on the partitions of the datasets and the configuration for model training.

**Performance metrics.** Mode performance is quantified using volumetric, topological, and distance scores. The volumetric scores include intersection-over-union (IoU), accuracy (ACC), centerlineDice (clDice), and AUC. For the topological score, the Betti Error β for the sum of Betti Numbers $\beta_{0}$ and $\beta_{1}$ is calculated. In the evaluation of DRIVE and STARE, β Error represents only $\beta_{1}$. For the distance score, Hausdorff Distance (HD) is used to quantify the accuracy of the boundary delineation and spatial closeness of the predicted and ground truth boundaries. In this study, IoU, ACC, AUC, and clDice are expressed as percentages (%). The Hausdorff Distance (HD) is measured in pixels (px).

### 4.2 Ablation optimization of preprocessing methods and loss functions

We selected U-Net as our baseline to assess how various preprocessing methods and loss functions affect its performance on MITO, ER, and the retinal blood vessel DRIVE datasets via ablation studies. Detailed results are shown in the Supplementary Material.

**Ablation optimization of preprocessing methods.** We test four methods (CLAHE, STD, Edge, Inversion, Fig. 3) with BCE loss on U-Net. Results show that CLAHE consistently enhances segmentation performance. Specifically, it increases IoU from 77.03% to 77.79% on the MITO dataset, from 74.61% to 75.26% on the ER dataset, and from 71.25% to 71.74% on the DRIVE dataset. STD further enhances IoU to 78.70% on the MITO dataset. One possible reason is that the mitochondrial networks are thicker than the ER and the blood vessel networks, indicating that it needs standardization for further scaling of foreground pixels. In contrast, edge enhancement causes substantial performance deterioration. Based on these results, we choose CLAHE for the ER and DRIVE datasets, and CLAHE with STD for the MITO dataset.

**Ablation optimization of loss functions.** Based on the previous experiments, we choose CLAHE or CLAHE with STD as our preprocessing method and examine segmentation performance under five different loss functions. We find Soft IOU consistently provides the best segmentation results. For example, it increases IoU from 78.80% under BCE to 79.80% on MITO and from 75.26% under BCE to 75.74% on ER.

In summary, the preprocessing method of CLAHE and the loss function of Soft IOU give overall the best performance for the models tested.

### 4.3 Influence of low-level features and topological self-similarity

We examine how *low-level features* and *topological self-similarity* influence the performance of the original U-Net in the segmentation of the organelle networks. Specifically, the performance of a standard U-Net with 4 down-sampling layers on 256×256 images is set as a baseline. Because a higher order of down-sampling can be viewed as extracting more high-level semantic features, we vary the depth of down-sampling layers from 4 to 1 to check whether *low-level features* are sufficient for segmentation. To check whether different resolutions of the organelle networks share similar topology information, we fix the U-Net configuration and vary the resolution of the input images by randomly cropping images ranging from 256 × 256 to 32×32. Then we test on 256×256 images. IoU metrics on the ER dataset and the MITO dataset are shown in Table 1.

**Table 1.** Segmentation performance of U-Net on ER and MITO.^a^

			**IoU** ↑	
Characteristics	Input	Down-sampling	ER	MITO
Low-level features	256	1	75.53	79.16
		2	75.48	**79.96**
		3	75.57	79.61
		4	**75.74**	79.80
Topological self-similarity	256	4	**75.74**	79.80
	128		75.26	**79.91**
	64		75.13	79.55
	32		74.98	78.63

a Bold numbers indicate the best performance.

When we reduce the depth of down-sampling from 4 to 1, the segmentation performance changes only slightly. For example, IoU decreases from 75.74% to 75.53% on ER (Table 1). This indicates that the contribution of high-level semantic features to segmentation is very small. When we reduce the resolution of input images, the segmentation performance also shows no substantial decline. For example, IoU decreases from 79.80% to 78.63% on MITO (Table 1). This indicates that organelle networks at different resolutions share similar topological information and provide similar features for segmentation.

### 4.4 Performance evaluation of MRE and $L_{hf}$

We conduct a comprehensive comparative analysis of several state-of-the-art (SOTA) segmentation networks to evaluate the performance of MRE and $L_{hf}$. The selection encompasses convolution-based architectures such as U-Net (Ronneberger *et al.* 2015), U-Net++ (Zhou *et al.* 2019), nnU-Net (Isensee *et al.* 2021), and HR-Net (Wang *et al.* 2020). Furthermore, Transformer-based models including PVT(Wang *et al.* 2021), UT-Net (Gao *et al.* 2021), TransUNet (Chen *et al.* 2021), and MedT (Valanarasu *et al.* 2021) are also included in the comparison. In addition, the recently introduced Segment Anything Model (SAM) (Kirillov *et al.* 2023) is included in the analysis. SAM is fine-tuned on the corresponding datasets to obtain segmentation without prompts. To assess the generalization of MRE, we incorporate it into U-Net, HR-Net, PVT and TransUNet. For a fair comparison, the PVT-Tiny model employed in this study has a comparable number of parameters and floating point operations (FLOPs) to U-Net.

**Figure 5.**
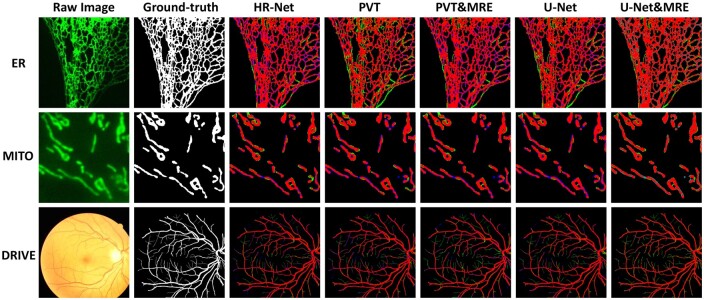
Comparison of segmentation results. Red: true positive. Green: false negative. Blue: false positive.

From the results of Table 2 and Fig. 5, we make the following observations. Firstly, incorporation of MRE enhances performance of U-Net, HR-Net, PVT and TransUNet across four datasets. Notably, PVT&MRE achieves significant performance improvement, with IoU score increases of 1.35%, 1.84%, 4.44%, and 6.26% on the ER, MITO, DRIVE, and STARE datasets, respectively.

**Table 2.** Performance metrics.^a^

		IoU↑	clDice↑	ACC↑	AUC↑	β Error↓	HD↓	IoU↑	clDice↑	ACC↑	AUC↑	β Error↓	HD↓
			
Model	Loss	ER						MITO					
SAM (Kirillov *et al.* 2023)	$L_{\mathrm{iou}}$	41.41	61.30	80.19	70.59	1309.00	9.65	49.11	67.42	94.52	81.54	400.00	6.11
UT-Net (Gao *et al.* 2021)	$L_{\mathrm{iou}}$	75.81	95.08	91.58	96.50	27.84	6.91	78.01	95.75	97.99	99.51	4.10	5.14
MedT (Valanarasu *et al.* 2021)	$L_{\mathrm{iou}}$	75.80	94.75	91.71	97.10	28.86	6.90	79.25	97.54	98.12	99.44	3.20	4.21
PVT (Wang *et al.* 2021)	$L_{\mathrm{iou}}$	75.12	94.20	91.30	96.91	32.79	6.97	78.36	96.41	98.01	99.56	2.80	4.36
TransUNet (Chen *et al.* 2021)	$L_{\mathrm{iou}}$	75.64	94.75	91.35	97.11	25.24	7.05	79.42	97.46	98.13	99.47	*2.70*	4.22
PVT&MRE	$L_{\mathrm{iou}}$	76.47	94.94	91.79	97.42	24.39	6.85	80.20	97.03	98.22	99.63	3.00	4.15
PVT&MRE	$L_{hf}$	76.74	*95.37*	91.93	97.50	**19.26**	*6.82*	80.61	97.15	98.26	99.66	*2.70*	4.18
TU&MRE	$L_{\mathrm{iou}}$	76.51	95.13	91.83	97.53	20.15	6.84	79.80	96.93	98.21	99.61	3.20	4.66
TU&MRE	$L_{hf}$	76.76	95.25	**91.98**	*97.57*	20.14	**6.76**	80.21	*97.71*	98.23	99.62	2.80	**3.96**
U-Net++ (Zhou *et al.* 2019)	$L_{\mathrm{iou}}$	75.45	94.67	91.51	97.40	26.03	6.99	79.48	97.33	98.20	99.55	**2.40**	4.24
nnU-Net (Isensee *et al.* 2021)	$L_{\mathrm{iou}}$	72.07	94.26	91.01	86.94	30.55	7.04	78.85	97.16	98.07	94.41	3.60	4.39
U-Net (Ronneberger *et al.* 2015)	$L_{\mathrm{iou}}$	75.74	94.69	91.65	97.38	28.73	6.87	79.80	97.09	98.10	99.60	2.80	4.56
HR-Net (Wang *et al.* 2020)	$L_{\mathrm{iou}}$	75.85	95.03	91.66	97.42	22.56	6.91	79.62	97.53	98.18	99.61	2.90	4.26
U-Net&MRE	$L_{\mathrm{iou}}$	76.47	95.12	91.83	97.48	20.97	*6.82*	80.31	97.32	98.24	99.60	2.90	4.21
U-Net&MRE	$L_{hf}$	**77.03**	**95.64**	*91.97*	*97.57*	*19.94*	*6.82*	**81.21**	97.62	**98.31**	**99.68**	2.80	*4.09*
HR-Net&MRE	$L_{\mathrm{iou}}$	76.71	95.22	91.96	*97.57*	22.01	6.83	80.63	97.45	98.26	99.64	3.10	4.13
HR-Net&MRE	$L_{hf}$	*76.92*	95.20	**91.98**	**97.63**	20.55	**6.76**	*80.70*	**97.73**	*98.27*	*99.67*	*2.70*	4.11
		**DRIVE**						**STARE**					
SAM (Kirillov *et al.* 2023)	$L_{\mathrm{iou}}$	48.23	60.28	94.33	75.93	446.00	7.07	46.41	59.25	92.02	74.31	452.00	7.96
UT-Net (Gao *et al.* 2021)	$L_{\mathrm{iou}}$	72.11	82.21	96.80	98.17	3.48	5.51	66.87	75.54	94.64	95.02	3.35	6.63
MedT (Valanarasu *et al.* 2021)	$L_{\mathrm{iou}}$	70.14	81.05	96.50	97.59	3.30	5.72	62.75	71.61	93.95	95.07	5.55	6.77
PVT (Wang *et al.* 2021)	$L_{\mathrm{iou}}$	68.49	78.89	96.30	97.80	3.73	5.80	61.64	71.11	93.74	94.74	3.65	6.78
TransUNet (Chen *et al.* 2021)	$L_{\mathrm{iou}}$	71.57	82.37	96.72	98.07	3.23	5.57	66.84	74.40	94.69	95.51	5.02	6.68
PVT&MRE	$L_{\mathrm{iou}}$	72.93	82.77	96.91	98.27	3.27	5.44	67.90	75.59	94.86	96.44	3.20	6.39
PVT&MRE	$L_{hf}$	73.35	82.92	96.96	98.45	2.85	5.39	68.49	76.47	94.99	96.53	3.12	6.38
TU&MRE	$L_{\mathrm{iou}}$	72.82	82.97	96.91	98.08	2.75	5.33	68.88	77.67	94.92	95.89	2.95	6.43
TU&MRE	$L_{hf}$	73.35	83.34	96.99	98.41	2.78	5.36	69.47	77.94	95.15	96.34	3.07	6.33
U-Net++ (Zhou *et al.* 2019)	$L_{\mathrm{iou}}$	73.30	83.25	96.95	*98.66*	2.88	5.40	67.49	76.65	94.53	96.56	3.27	6.54
nnU-Net (Isensee *et al.* 2021)	$L_{\mathrm{iou}}$	72.60	82.42	96.86	90.91	4.00	5.41	69.42	76.94	95.20	87.06	2.60	6.38
U-Net (Ronneberger *et al.* 2015)	$L_{\mathrm{iou}}$	73.24	83.04	96.92	98.61	2.95	5.41	67.71	75.97	94.93	96.28	3.23	6.63
HR-Net (Wang *et al.* 2020)	$L_{\mathrm{iou}}$	72.86	83.29	96.87	98.53	2.83	5.41	69.33	76.88	95.11	96.65	2.74	6.38
U-Net&MRE	$L_{\mathrm{iou}}$	73.71	83.53	97.01	98.65	2.66	5.34	69.36	76.72	95.12	96.95	3.00	6.43
U-Net&MRE	$L_{hf}$	*74.05*	*83.62*	**97.04**	98.72	*2.64*	*5.31*	*70.07*	78.09	95.24	**97.10**	*2.50*	6.36
HR-Net&MRE	$L_{\mathrm{iou}}$	73.85	83.44	*97.02*	*98.73*	2.83	5.35	70.02	*78.21*	*95.25*	97.02	2.87	*6.30*
HR-Net&MRE	$L_{hf}$	**74.08**	**83.83**	**97.04**	**98.78**	**2.57**	**5.30**	**70.16**	**78.62**	**95.27**	*97.03*	**2.45**	**6.23**

a Bold numbers indicate the best performance and italicized numbers represent the next best performance.

Secondly, $L_{hf}$ leads to further performance improvement. With both MRE and $L_{hf}$, U-Net achieved leading performance on the ER and MITO datasets, with IoU improved by 1.18% and 1.41% compared to prior state-of-the-art models. Similarly, HR-Net with MRE and $L_{hf}$ lead on the STARE and DRIVE datasets, with IoU improved by 0.84% and 0.74%. These results confirms the effectiveness of MRE and $L_{hf}$.

Thirdly, Transformer-based models often underperform convolution-based models in the segmentation of biological and medical images. However, with the assistance of MRE and $L_{hf}$, the performance of PVT and TransUNet (TU) has surpassed that of prior models on the four datasets.

Lastly, the high performance of U-Net and HR-Net on the DRIVE and STARE datasets confirms the generalization capability of these models on different complex network structures. The MRE combined with the $L_{hf}$ has demonstrated potential across various models and diverse segmentation tasks.

### 4.5 Ablation study

**Loss function.** We compare different losses with U-Net and PVT. *Single loss* means only the top layer (j=0) prediction-label pair is used to compute loss. *Up-sampling loss* means only $L_{up}$ is used to compute loss. *Multi-layer loss* means only $L_{ml}$ is used to compute loss. *Hierarchical fusion loss* $L_{hf}$ combines *up-sampling loss* with *multi-layer loss*. Experimental results on the ER, MITO, DRIVE, and STARE datasets are shown in Table 3. We can observe that compared to the $L_{\mathrm{single}}$, both $L_{up}$ and $L_{ml}$ contribute to enhanced performance for PVT&MRE and U-Net&MRE. Moreover, the *hierarchical fusing loss*, which integrates $L_{up}$ and $L_{ml}$, achieves the best segmentation performance in the majority of cases.

**Table 3.** Segmentation performance of different loss functions.^a^

Loss	U-Net&MRE				PVT&MRE			
	ER	MITO	DRIVE	STARE	ER	MITO	DRIVE	STARE
$L_{\mathrm{single}}$	76.47	80.31	73.71	69.36	76.47	80.20	72.93	67.90
$L_{up}$	76.78	80.50	73.64	**70.19**	**76.78**	**80.68**	73.27	68.17
$L_{ml}$	76.60	80.56	**74.05**	69.52	76.63	80.58	72.99	68.25
$L_{hf}$	**77.03**	**81.21**	**74.05**	70.07	76.74	80.61	**73.35**	**68.49**

a Bold numbers indicate the best performance.

**Random cropping.** Because random cropping is used in model inference, we examine the influence of the randomness on model performance by repeating the same inference experiments using PVT&MRE and U-Net&MRE on the four datasets (ER, MITO, DRIVE, START) ten times using different random seeds. The results show that the influence is very small. See Supplementary Fig. S1 and Supplementary Table S3 for further details.

### 4.6 Topological and morphological analysis

**Figure 6.**
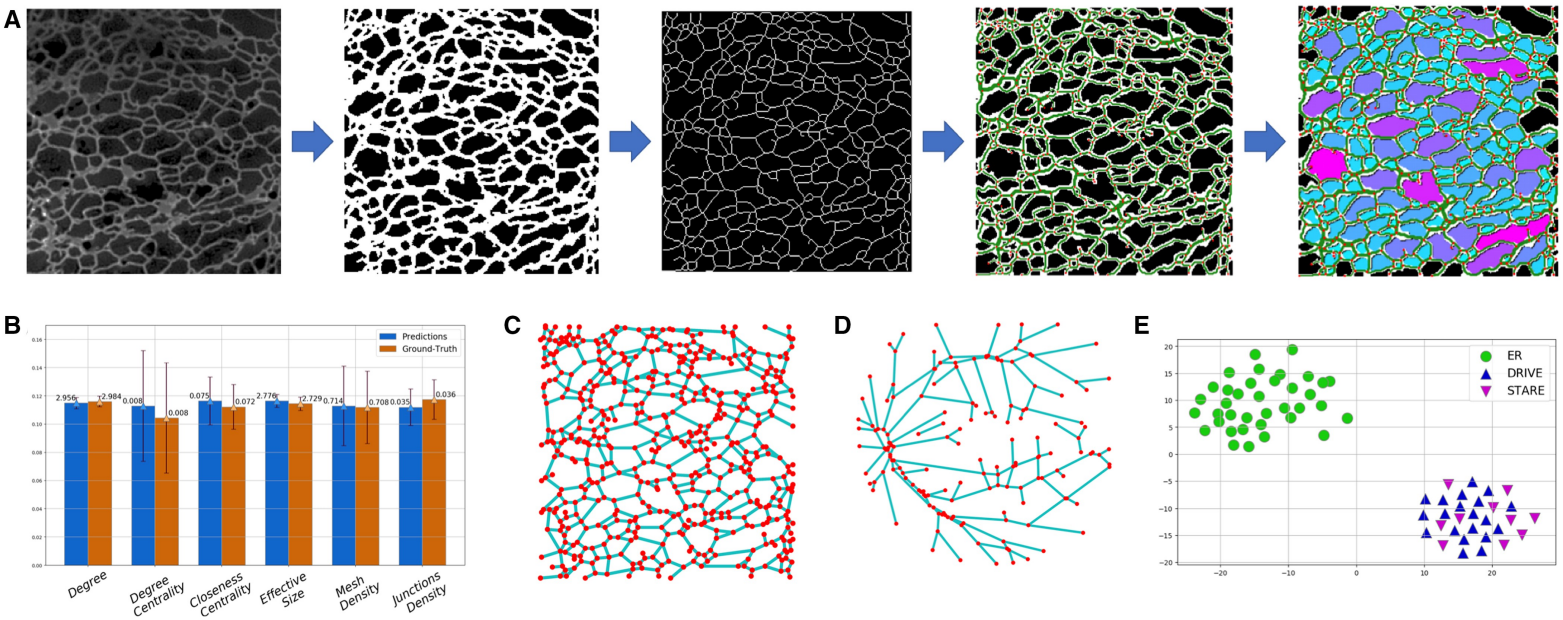
Analysis and comparison of network topology. (A) Analysis pipeline. In column 4, red dots and green lines denote nodes (junctions) and edges of the graph constructed, respectively. In column 5, colors indicate mesh areas. A warmer color indicates a larger mesh area. (B) Topological properties: segmentation results (*n* = 38) versus ground truth (*n* = 38). Bars are rescaled for convenience of comparison. Error bars indicate SD (standard deviation). *P*-values of comparison using two-sample *t*-tests: 0.13, 0.29, 0.24, 0.27, 0.90, 0.17. (C and D) Representative graph representation of an ER network (C) versus a retinal vessel network (D). (E) Comparison of topological properties of ER networks (*n* = 38) versus retinal blood vessel networks (*n* = 30).

Topological properties define how a network is connected internally. From the segmentation result of an ER network, its graph representation is constructed. Its topological properties are then calculated from the graph representation. Figure 6A outlines the analysis pipeline. First, U-Net with MRE is used to segment the ER network to extract its morphology. Small and isolated segmentation blobs that result from background noise are removed. Second, a one-pixel-wide skeleton is extracted from the segmentation result (Lee *et al.* 1994). Third, a graph construction algorithm is adapted to scan the skeleton to detect its junctions and tubules, which are represented as nodes and edges in the graph, respectively (Wang *et al.* 2018). We define a junction as a pixel associated with either one terminal or more than two branches. Fourth, four common junction properties, namely *degree*, *degree centrality*, *closeness centrality*, and *effective size* (Newman 2008), as well as two customized network properties, namely *mesh density* and *junction density*, are calculated from the graph constructed. These properties are defined as follows:

*Definition 1:* The *degree* of a junction is the number of edges with which it is associated or the number of junctions with which it is directly connected.

*Definition 2:* The *degree centrality* of a junction is its degree normalized by the maximum possible degree of the network, i.e. the total number of junctions.

*Definition 3:* The *closeness centrality* of a junction is its mean shortest path to all the other junctions. It characterizes how close a junction is to the others.

*Definition 4:* The *effective size* of a junction is the number of its directly connected junctions with which no common neighbors are shared. It characterizes the non-redundancy of a junction.

*Definition 5:* The *mesh density* of a network is the ratio between the area of all loops enclosed by its edges and the area of the convex hull confined by the network.

*Definition 6:* The *junction density* of a network is the ratio between the total number of junctions and the total length of all edges. The mesh density and the junction density can be used to characterize the efficiency of cargo transport within a physical network such as the ER network along its junctions.

The first four network properties are computed for each junction of a network. These attributes are averaged over all junctions to obtain a network-wide metric. To benchmark the performance of our analysis pipeline, we compare the network property metrics computed from our segmentation results against those computed from the ground truth (Fig. 6B). The comparison confirms that our segmentation is sufficiently accurate to support topological analysis of the ER network.

To further test our network property analysis, we use it to examine the topological properties of retinal blood vessel networks in the DRIVE and STARE datasets. Figure 6C and D visually compare the reconstructed graph representation of an ER network versus that of a retinal blood vessel network from the DRIVE dataset. We then used t-SNE (Van der Maaten and Hinton 2008) to compare the extracted network properties (Fig. 6E). The comparison shows that the ER network differs significantly from the retinal blood vessel network, in qualitative agreement with the visual comparison.

Accurate segmentation of the ER network enables the study of its dynamic topological properties in live cells. We have also used our models for segmenting videos of the ER network. We find that, compared to U-Net, U-Net&MRE shows more consistent segmentation results. Furthermore, the latency incurred by the calculation of MRE and $L_{hf}$ is very small. Please refer to the Supplementary Material for further details.

## 5 Conclusion

Our study develops a multi-resolution encoder (MRE) and a hierarchical fusion loss (Lhf) tailored for segmenting organelle networks from fluorescence microscopy images. Utilizing these components, we achieved superior performance over state-of-the-art models in segmenting both organelle and retinal blood vessel networks, indicating strong generalization capability in complex network segmentation. The construction and refinement of MRE and Lhf leveraged the distinct properties of organelle networks and microscopy imaging to optimize feature extraction and semantic segmentation.

While fluorescence microscopy is important to biological research, it poses distinctive challenges for image processing, which we have addressed by adapting deep learning models to the specific characteristics of these images. This approach not only enhances segmentation accuracy but also facilitates morphological and topological analyses, providing deeper insights into organelle dynamics.

Our study has its limitations. The computational demands of MRE are nontrivial, and further validation across a broader range of organelle networks is necessary. Our segmentation models are designed to segment individual images and, therefore, do not consider the spatial and temporal continuity between video frames. However, the improvements realized in segmentation performance and analytic precision are promising, offering a refined method for detailed study of intracellular structures.

## Author contributions

Ablation optimization: Y.L., Y.G.; data acquisition: W.L., Y.L.; architecture design: J.H., Y.L., G.Y.; comparative experiment: J.H., Z.W.; interpretation of results and ablation study: J.H., Y.L.; morphological and topological analysis: Y.L., Y.G., W.L., J.H.; open sourcing: J.H., Z.W., G.Y.; drafting the manuscript: J.H., Y.L., Y.G., G.L. and G.Y.

## Supplementary Material

btae559_Supplementary_Data
