# Supplementary material for: Accurate segmentation of intracellular organelle networks using low-level features and topological self-similarity
Source: Bioinformatics. 2024 Sep 20;40(10):btae559. doi: 10.1093/bioinformatics/btae559 (PMC11467052; doi:10.1093/bioinformatics/btae559)
Supplement: btae559_Supplementary_Data [file btae559_supplementary_data.zip › SupplementaryMaterial_Final.docx]

**Supplementary Material**

1. Construction of custom datasets

To develop deep learning segmentation models for organelle networks, we construct two custom image datasets: ER and MITO, for the ER network and the mitochondrial network, respectively. Fluorescence microscopy images of the ER network and mitochondrial network in live cells are acquired using spinning disk confocal microscopy and widefield microscopy, respectively. In this way, the datasets are representative of the imaging conditions commonly used in related biomedical studies. Detailed descriptions of experimental procedures are given at IEEE DataPort (DOI: 10.21227/t2he-zn97).

The acquired images are manually annotated by experts using ITK-SNAP to produce binary masks as ground truth. Annotated images are partitioned into a training set, a validation set, and a test set. All images are cropped into 256×256 patches with overlap for training and without overlap for validation and testing. Horizontal and vertical flipping as well as 90°/180°/270° rotation are used for data augmentation. To mimic actual experimental conditions, different levels of Gaussian noise are added to the training images for additional data augmentation. Mean and standard deviation of the added noise are drawn randomly from the actual range of these parameters in real experimental images, making a final typical signal-to-noise ratio of approximately 4.

1. Ablation optimization of preprocessing methods and loss functions

Preprocessing Methods: Four methods are tested: equalization (CLAHE), edge enhancement, standardization and inversion. Biological fluorescence microscopy images have high bit-depths and large dynamic ranges, which pose a challenge to models developed for low bit-depths natural images. To address this, equalization and standardization are employed. The blurring of object boundaries is another distinct property of fluorescence microscopy images. We mitigate this problem using edge enhancement. Inversion of bright-field images into dark-field images is commonly used in fluorescence microscopy for visualization of thin structures. We test it as a preprocessing method.

Loss Functions: Given an image $\boldsymbol{I}$ with $\boldsymbol{N}$ pixels, we denote its segmentation ground truth as $\boldsymbol{y}$, in which foreground and background pixels are labeled 1 and 0, respectively. We denote the prediction of a segmentation model as $\hat{\mathbf{y}}\boldsymbol{\in}\left[ \mathbf{0,1} \right]$, which represents probabilities of individual pixels classified as foreground.

BCE loss measures the total amount of information from the prediction of a model. A low BCE indicates a good fit of the prediction with the ground truth. The following formulation is used:

$$\begin{aligned} \mathcal{L}_{\mathcal{bce}}\boldsymbol{=-}\frac{\boldsymbol{1}}{\boldsymbol{N}}\sum_{\boldsymbol{i=1}}^{\boldsymbol{N}} \boldsymbol{y}_{\boldsymbol{i}}\boldsymbol{\cdot}\log\left( \hat{\boldsymbol{y}_{\boldsymbol{i}}} \right)\boldsymbol{+}\left( \boldsymbol{1-}\boldsymbol{y}_{\boldsymbol{i}} \right)\boldsymbol{\cdot}\log\left( \boldsymbol{1-}\hat{\boldsymbol{y}_{\boldsymbol{i}}} \right)\boldsymbol{\#}\left( \boldsymbol{1} \right) \end{aligned}$$

The imbalance between different label classes is not considered in BCE loss. Overwhelming of minority classes by majority classes leads to model bias. WBCE is a variant of BCE that assigns a weight $\boldsymbol{w}_{\boldsymbol{i}}$ to each class to reweight *a priori* its contribution to the total loss. The following formulation is used:

$$\begin{aligned} \mathcal{L}_{\mathcal{wbce}}\boldsymbol{=-}\frac{\boldsymbol{1}}{\boldsymbol{N}}\sum_{\boldsymbol{i=1}}^{\boldsymbol{N}} \boldsymbol{w}_{\boldsymbol{1}}\boldsymbol{y}_{\boldsymbol{i}}\boldsymbol{\cdot}\log\left( \hat{\boldsymbol{y}_{\boldsymbol{i}}} \right)\boldsymbol{+}\boldsymbol{w}_{\boldsymbol{2}}\left( \boldsymbol{1-}\boldsymbol{y}_{\boldsymbol{i}} \right)\boldsymbol{\cdot}\log\left( \boldsymbol{1-}\hat{\boldsymbol{y}_{\boldsymbol{i}}} \right)\boldsymbol{\#}\left( \boldsymbol{2} \right) \end{aligned}$$

Intersection over union (IoU) is a commonly adopted metric of segmentation accuracy. The following differentiable Soft IOU is used:

$$\begin{aligned} \mathcal{L}_{\mathcal{iou}}\boldsymbol{=1-}\frac{\sum_{\boldsymbol{i=1}}^{\boldsymbol{N}} \boldsymbol{y}_{\boldsymbol{i}}\hat{\boldsymbol{y}_{\boldsymbol{i}}}}{\sum_{\boldsymbol{i=1}}^{\boldsymbol{N}} \left( \boldsymbol{y}_{\boldsymbol{i}}\boldsymbol{+}\hat{\boldsymbol{y}_{\boldsymbol{i}}}\boldsymbol{-}\boldsymbol{y}_{\boldsymbol{i}}\hat{\boldsymbol{y}_{\boldsymbol{i}}} \right)\boldsymbol{+}\boldsymbol{\eta}}\boldsymbol{,\#}\left( \boldsymbol{3} \right) \end{aligned}$$

where $\boldsymbol{\eta}$is a smoothing coefficient to prevent the denominator from becoming zero. Predictions that indicate the likelihood of individual pixels belonging to the foreground are used to calculate this loss.

The Dice Coefficient is another commonly used loss function to evaluate the performance of a segmentation model. Similar to Soft IOU, it also focuses on the overlap between the prediction and the ground truth. It correlates positively with the Soft IOU but is rescaled. The following formulation is used:

$$\begin{aligned} \mathcal{L}_{\mathcal{dice}}\boldsymbol{=1-}\frac{\boldsymbol{2}\sum_{\boldsymbol{i=1}}^{\boldsymbol{N}} \boldsymbol{y}_{\boldsymbol{i}}\hat{\boldsymbol{y}_{\boldsymbol{i}}}}{\sum_{\boldsymbol{i=1}}^{\boldsymbol{N}} \left( \boldsymbol{y}_{\boldsymbol{i}}\boldsymbol{+}\hat{\boldsymbol{y}_{\boldsymbol{i}}} \right)\boldsymbol{+}\boldsymbol{\eta}}\boldsymbol{\#}\left( \boldsymbol{4} \right) \end{aligned}$$

Focal Loss originally aimed to solve the data imbalance problem commonly encountered in object detection. It penalizes “easy background” examples that barely help in training and forces the model to focus on learning hard negative examples. The following formulation is used:

$$\begin{aligned} \mathcal{L}_{\mathcal{focal}}\boldsymbol{=-}\frac{\boldsymbol{1}}{\boldsymbol{N}}\sum_{\boldsymbol{i=1}}^{\boldsymbol{N}} \left( \boldsymbol{1-}\boldsymbol{\alpha} \right)\left( \boldsymbol{1-}\boldsymbol{y}_{\boldsymbol{i}} \right)\hat{{\boldsymbol{y}_{\boldsymbol{i}}}^{\boldsymbol{\gamma}}}\log\left( \boldsymbol{1-}\hat{\boldsymbol{y}_{\boldsymbol{i}}} \right)\boldsymbol{+a}\boldsymbol{y}_{\boldsymbol{i}}\left( \boldsymbol{1-}\hat{\boldsymbol{y}_{\boldsymbol{i}}} \right)^{\boldsymbol{\gamma}}\log\left( \hat{\boldsymbol{y}_{\boldsymbol{i}}} \right)\boldsymbol{,}\boldsymbol{\#}\left( \boldsymbol{5} \right) \end{aligned}$$

where $\boldsymbol{\alpha}\boldsymbol{\in}\left[ \mathbf{0,1} \right]$ $\boldsymbol{\gamma}\mathbf{> 0}$ . Focal Loss is similar to WBCE in conditionally reweighting model predictions.

1. Complete list of performance metrics of U-Net under different preprocessing methods and loss functions.

**Table S1.** Performance under different preprocessing methods & loss functions.

| **Model** | **Condition** | **Dataset** | **Item** | **IoU** | **F1** | **AUC** | **SPEC** | **SEN** | **ACC** |
| --- | --- | --- | --- | --- | --- | --- | --- | --- | --- |
| **U-Net** | **Prepro**  (Loss=BCE) | **MITO** | None | 77.03 | 87.03 | 99.53 | 98.62 | 89.23 | 97.87 |
|  |  |  | CLAHE | 77.79 | 87.51 | 99.55 | **98.81** | 88.42 | 97.98 |
|  |  |  | STD | **78.70** | **88.08** | **99.60** | 98.77 | **89.84** | **98.05** |
|  |  |  | Inversion | 78.23 | 87.79 | 99.56 | 98.75 | 89.48 | 98.01 |
|  |  |  | Edge | 70.06 | 82.40 | 99.16 | 97.82 | 87.64 | 97.00 |
|  |  | **ER** | None | 74.61 | 85.46 | 96.36 | 92.87 | 86.86 | 91.05 |
|  |  |  | CLAHE | **75.26** | **85.89** | **97.09** | **93.29** | **86.89** | **91.35** |
|  |  |  | STD | 74.77 | 85.57 | 96.85 | 93.22 | 86.45 | 91.17 |
|  |  |  | Inversion | 74.98 | 85.70 | 96.47 | 93.19 | 86.73 | 91.23 |
|  |  |  | Edge | 69.56 | 82.05 | 94.71 | 90.88 | 84.17 | 88.85 |
|  |  | **DRIVE** | None | 71.25 | 83.21 | 98.42 | 98.23 | 82.63 | 96.67 |
|  |  |  | CLAHE | **71.74** | **83.54** | **98.45** | 98.26 | 82.98 | **96.74** |
|  |  |  | STD | 71.09 | 83.10 | 98.21 | **98.33** | 81.79 | 96.68 |
|  |  |  | Inversion | 71.20 | 83.18 | 98.42 | 98.16 | **83.03** | 96.65 |
|  |  |  | Edge | 71.09 | 83.10 | 98.21 | **98.33** | 81.79 | 96.68 |
|  | **Loss** | **MITO**  (Prepro=  CLAHE+STD) | BCE | 78.80 | 88.14 | 99.60 | 98.75 | 90.11 | 98.06 |
|  |  |  | WBCE | 77.77 | 87.49 | 99.55 | 98.86 | 87.93 | 97.99 |
|  |  |  | IOU | **79.80** | **88.77** | **99.62** | 98.87 | **90.19** | **98.17** |
|  |  |  | DICE | 79.25 | 88.42 | 99.34 | **98.88** | 89.45 | 98.12 |
|  |  |  | FOCAL | 71.05 | 83.07 | 99.14 | 97.95 | 87.78 | 97.14 |
|  |  | **ER**  (Prepro=  CLAHE) | BCE | 75.26 | 85.89 | 97.09 | 93.29 | **86.89** | 91.35 |
|  |  |  | WBCE | 75.28 | 85.89 | 96.96 | 93.42 | 86.68 | 91.38 |
|  |  |  | IOU | **75.74** | **86.20** | **97.17** | **94.04** | 86.13 | **91.65** |
|  |  |  | DICE | 74.91 | 85.65 | 95.64 | 93.30 | 86.47 | 91.23 |
|  |  |  | FOCAL | 74.81 | 85.59 | 96.77 | 93.47 | 86.05 | 91.22 |
|  |  | **DRIVE**  (Prepro=  CLAHE) | BCE | 71.74 | 83.54 | 98.45 | 98.26 | 82.98 | 96.74 |
|  |  |  | WBCE | 69.90 | 82.29 | 98.26 | 98.24 | 81.02 | 96.52 |
|  |  |  | IOU | **73.24** | **84.55** | **98.65** | **98.29** | **84.50** | **96.92** |
|  |  |  | DICE | 72.53 | 84.08 | 96.44 | 98.25 | 84.00 | 96.82 |
|  |  |  | FOCAL | 66.10 | 79.59 | 97.44 | 97.91 | 78.59 | 95.98 |

1. Experimental setup

Detailed partitions of the four datasets: The DRIVE dataset contains 40 images (20 for training and 20 for testing) with a resolution of 565×584. The STARE dataset contains 20 images (10 for training and 10 for testing) with a resolution of 700×605. Both datasets are distributed publicly for the development and evaluation of deep learning models for the diagnosis of related eye diseases, such as central retinal artery occlusion. For each image in DRIVE and STARE, a binary mask from manual segmentation is provided. Similar as in the processing of ER and MITO images, we crop the images into 256×256 patches with overlap for training and without overlap for testing. The CLAHE preprocessing method is applied for both DRIVE and STARE.

**Table S2.** Detailed configurations of datasets.

| Dataset | Train | Train Augmentation | Valid | Test |
| --- | --- | --- | --- | --- |
| ER | 157 | 942 | 28 | 38 |
| MITO | 165 | 1980 | 20 | 10 |
| DRIVE | 272 | 1632 | 80 | 80 |
| STARE | 40 | 240 | 40 | 40 |

Configurations: All models are implemented using PyTorch (version 2.0.1) and run on 4 NVIDIA GeForce RTX 3090 GPU cards. During training, we first validate our configuration and then use the same training configurations for all models. Specifically, we use a standard SGD optimizer and set the initial learning rate as 0.01. We divide the learning rate by 10 for every 5 epochs and use 50 epochs in total. The batch size is set to 32 for the ER and MITO, and 16 for the DRIVE and STARE. The regularization weight is set as 0.0005. Parameters for the loss functions are set as follows: the weights in WBCE are determined adaptively for each image based on the fraction of foreground and background pixels in the ground truth, $\boldsymbol{\eta}\mathbf{=1}\boldsymbol{e}^{\mathbf{-10}}$ for Soft IOU, $\boldsymbol{\eta}\mathbf{=1}\boldsymbol{e}^{\mathbf{-10}}$ for Dice Coefficient, and $\boldsymbol{\alpha}\mathbf{=0.25}$, $\boldsymbol{\gamma}\mathbf{=2}$ for Focal Loss. Model inference is run on 1 NVIDIA GeForce RTX 3090 GPU card.

1. Characterizing influence of random cropping on model performance

Random Cropping. The cropped images were obtained by randomly cropping from the query image during the inference time. The cropping method is consistent with that used during the training phase. Specifically, our input consists of two components: one is the original image, which is fed into the model's encoder. The other component comprises multi-scale images, including $\boldsymbol{I}_{\boldsymbol{raw}}$, $\boldsymbol{I}_{\boldsymbol{1}}$, $\boldsymbol{I}_{\boldsymbol{2}}$, $\boldsymbol{I}_{\boldsymbol{3}}$, and $\boldsymbol{I}_{\boldsymbol{4}}$, where $\boldsymbol{I}_{\boldsymbol{raw}}$ is the original image, $\boldsymbol{I}_{\boldsymbol{1}}$ is derived from a random crop of the original image with a size that is half of $\boldsymbol{I}_{\boldsymbol{raw}}$; $\boldsymbol{I}_{\boldsymbol{2}}$ is obtained by randomly cropping $\boldsymbol{I}_{\boldsymbol{1}}$, with a size that is half of $\boldsymbol{I}_{\boldsymbol{1}}$; similarly, $\boldsymbol{I}_{\boldsymbol{2}}$ and $\boldsymbol{I}_{\boldsymbol{3}}$ follow the same principle. The images $\boldsymbol{I}_{\boldsymbol{raw}}$ through $\boldsymbol{I}_{\boldsymbol{4}}$ serve as inputs for the multi-resolution encoder (MRE) to capture low-level features at different scales, thereby enriching the model's sources of information. To validate the impact of random cropping on model performance, we conducted additional experiments by setting 10 different random seeds during the model inference. The results are presented in Figure S1 and Table S3. The experimental results indicate that the impact of different random seeds on the model inference results is minimal. This demonstrates the reliability and robustness of our models.

**
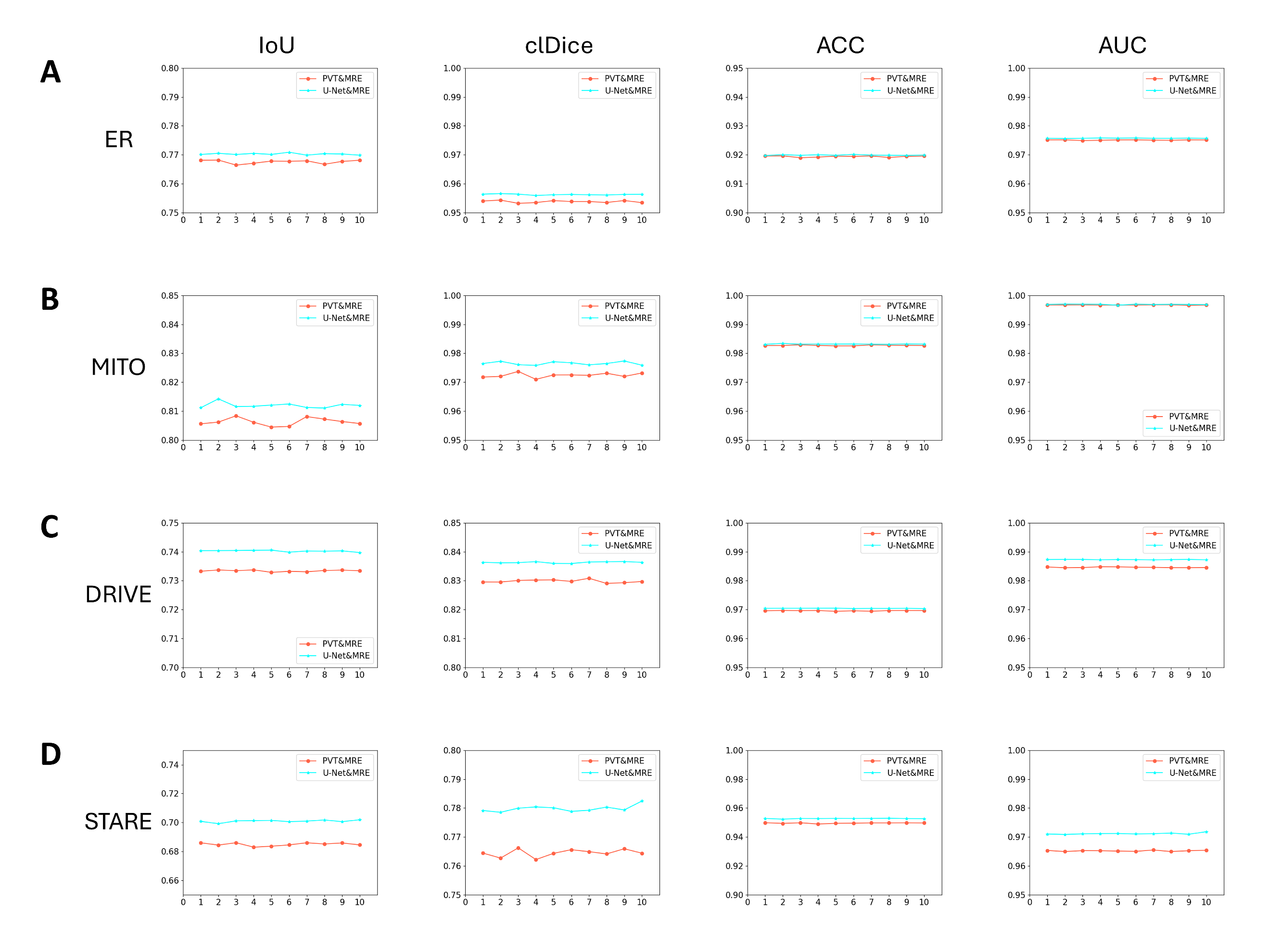
**

**Figure S1.** Assessing the influence of cropping randomness on model performance in inference. (A) Performance measured in IoU, clDice, ACC and AUC of PVT&MRE as well as U-Net&MRE on the ER dataset in ten experimental runs using different random seeds. X axis label: index of experimental runs. Y-axis label: performance metric. (B) Results of the same experiments as in (A) except on the MITO dataset. (C) Same as in (A) except on the DRIVE dataset. (D) Same as in (A) except on the STARE dataset.

**Table S3.** Quantifying influence of cropping randomness on model performance in inference using statistics of five performance metrics. Each model is tested on four different datasets in ten repeated experiments using different random seeds. Results shown in Figure S1 are generated from the same experiments. STD: standard deviation.

| Model | Dataset | Metric | Min | Max | Mean | STD | Model | Dataset | Metric | Min | Max | Mean | STD |
| --- | --- | --- | --- | --- | --- | --- | --- | --- | --- | --- | --- | --- | --- |
| PVT  &MRE | ER | IoU | 0.7664 | 0.7682 | 0.7676 | 0.0005 | **U-Net**  **&MRE** | ER | IoU | 0.7698 | 0.7709 | 0.7702 | 0.0003 |
|  |  | clDice | 0.9532 | 0.9543 | 0.9538 | 0.0003 |  |  | clDice | 0.9559 | 0.9566 | 0.9563 | 0.0001 |
|  |  | ACC | 0.9190 | 0.9196 | 0.9194 | 0.0002 |  |  | ACC | 0.9197 | 0.9201 | 0.9199 | 0.0001 |
|  |  | AUC | 0.9749 | 0.9751 | 0.9750 | 8e-05 |  |  | AUC | 0.9757 | 0.9758 | 0.9757 | 5e-05 |
|  |  | HD | 6.7725 | 6.8389 | 6.8017 | 0.0173 |  |  | HD | 6.7969 | 6.8273 | 6.8120 | 0.0090 |
|  | MITO | IoU | 0.8045 | 0.8084 | 0.8063 | 0.0012 |  | MITO | IoU | 0.8111 | 0.8143 | 0.8120 | 0.0008 |
|  |  | clDice | 0.9709 | 0.9737 | 0.9724 | 0.0007 |  |  | clDice | 0.9758 | 0.9773 | 0.9765 | 0.0005 |
|  |  | ACC | 0.9825 | 0.9829 | 0.9827 | 0.0001 |  |  | ACC | 0.9831 | 0.9834 | 0.9832 | 9e-05 |
|  |  | AUC | 0.9965 | 0.9967 | 0.9966 | 6e-05 |  |  | AUC | 0.9966 | 0.9969 | 0.9968 | 9e-05 |
|  |  | HD | 4.1641 | 4.2312 | 4.1834 | 0.0199 |  |  | HD | 3.9895 | 4.095 | 4.0555 | 0.0332 |
|  | DRIVE | IoU | 0.7328 | 0.7337 | 0.7333 | 0.0002 |  | DRIVE | IoU | 0.7397 | 0.7406 | 0.7403 | 0.0002 |
|  |  | clDice | 0.8291 | 0.8308 | 0.8298 | 0.0005 |  |  | clDice | 0.8359 | 0.8366 | 0.8363 | 0.0002 |
|  |  | ACC | 0.9694 | 0.9697 | 0.9696 | 9e-05 |  |  | ACC | 0.9703 | 0.9705 | 0.9704 | 4e-05 |
|  |  | AUC | 0.9844 | 0.9847 | 0.9846 | 0.0001 |  |  | AUC | 0.9871 | 0.9873 | 0.9872 | 5e-05 |
|  |  | HD | 5.3764 | 5.4132 | 5.3915 | 0.0108 |  |  | HD | 5.3093 | 5.3417 | 5.3267 | 0.0106 |
|  | STARE | IoU | 0.6830 | 0.9499 | 0.9496 | 0.0026 |  | STARE | IoU | 0.6992 | 0.7019 | 0.7009 | 0.0001 |
|  |  | clDice | 0.7622 | 0.7662 | 0.7645 | 0.0012 |  |  | clDice | 0.7785 | 0.7824 | 0.7798 | 0.0010 |
|  |  | ACC | 0.9489 | 0.9499 | 0.9496 | 0.0002 |  |  | ACC | 0.9524 | 0.9530 | 0.9528 | 0.0001 |
|  |  | AUC | 0.9649 | 0.9655 | 0.9652 | 0.0001 |  |  | AUC | 0.9708 | 0.9718 | 0.9711 | 0.0002 |
|  |  | HD | 6.3227 | 6.4070 | 6.3706 | 0.0250 |  |  | HD | 6.3372 | 6.4131 | 6.3639 | 0.0225 |

1. Topological analysis of the ER network.

Dynamic topological property: Accurate segmentation of the ER network also allows us to study its dynamic topological properties in live cells. Figure S2 A shows a local region within an ER network that centers around a lysosome, another intracellular organelle that moves along the ER network. After segmentation (Figure S2-B) and graph construction (Figure S2-C), we calculate the number of local junctions and edges within an analysis window of 9 pixels in radius. Figure S2-D shows that these two properties increase consistently over time, indicating that the region centered around the moving lysosome increases continuously in its complexity and density. This analysis of dynamic topological properties provides tools to investigate the relationship between the local organization of the ER network with the dynamic behavior of the lysosome. This type of analysis is essential to elucidating the interactions between the lysosome and the ER network.


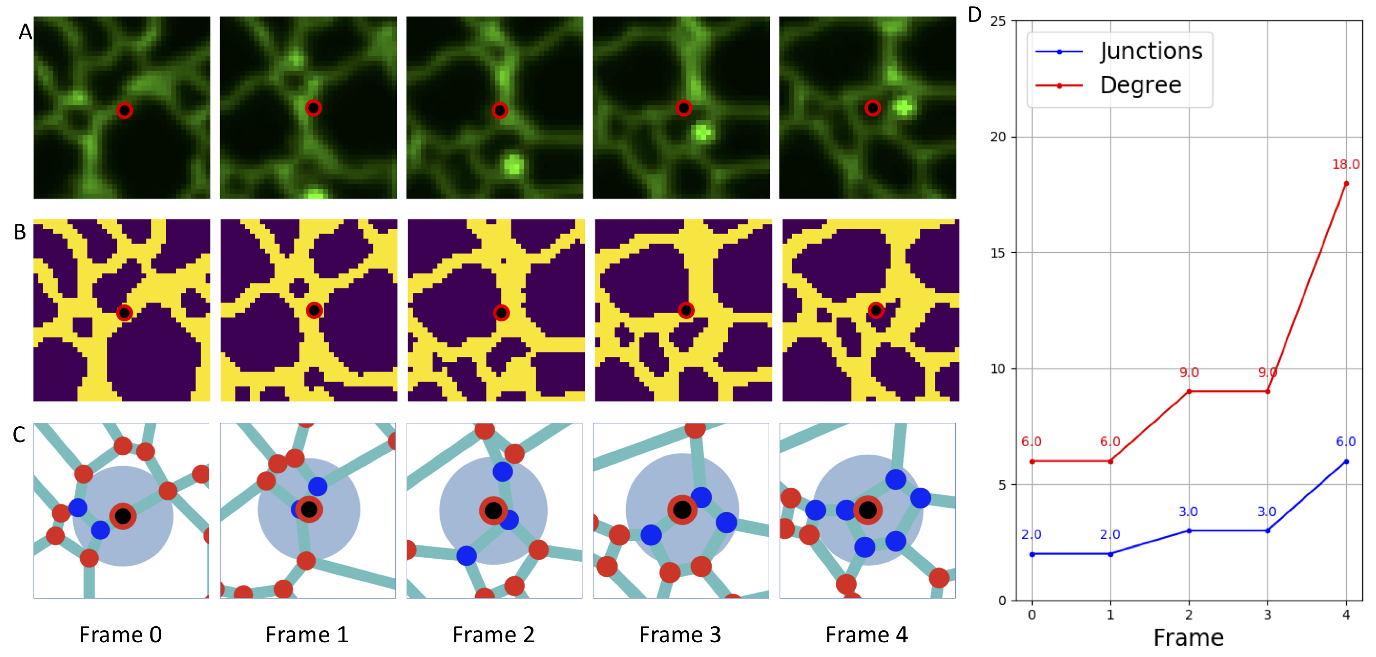


Figure S2. Analyzing dynamic topological properties of the ER network. (A) A lysosome's path along an ER network's tubules and junctions, with its position marked in red. (B) Segmentation of the ER network. (C) Graph representation of the ER network. Red dots and cyan lines denote junctions and tubules of the ER network, respectively. Red circle denotes the location of the lysosome. Blue disc denotes an analysis window with a radius of 9 pixels to quantify local network topological properties. (D) Dynamic topological property of the network is represented by the number of junctions (blue) and the number of local edges (red) within the analysis window over time.


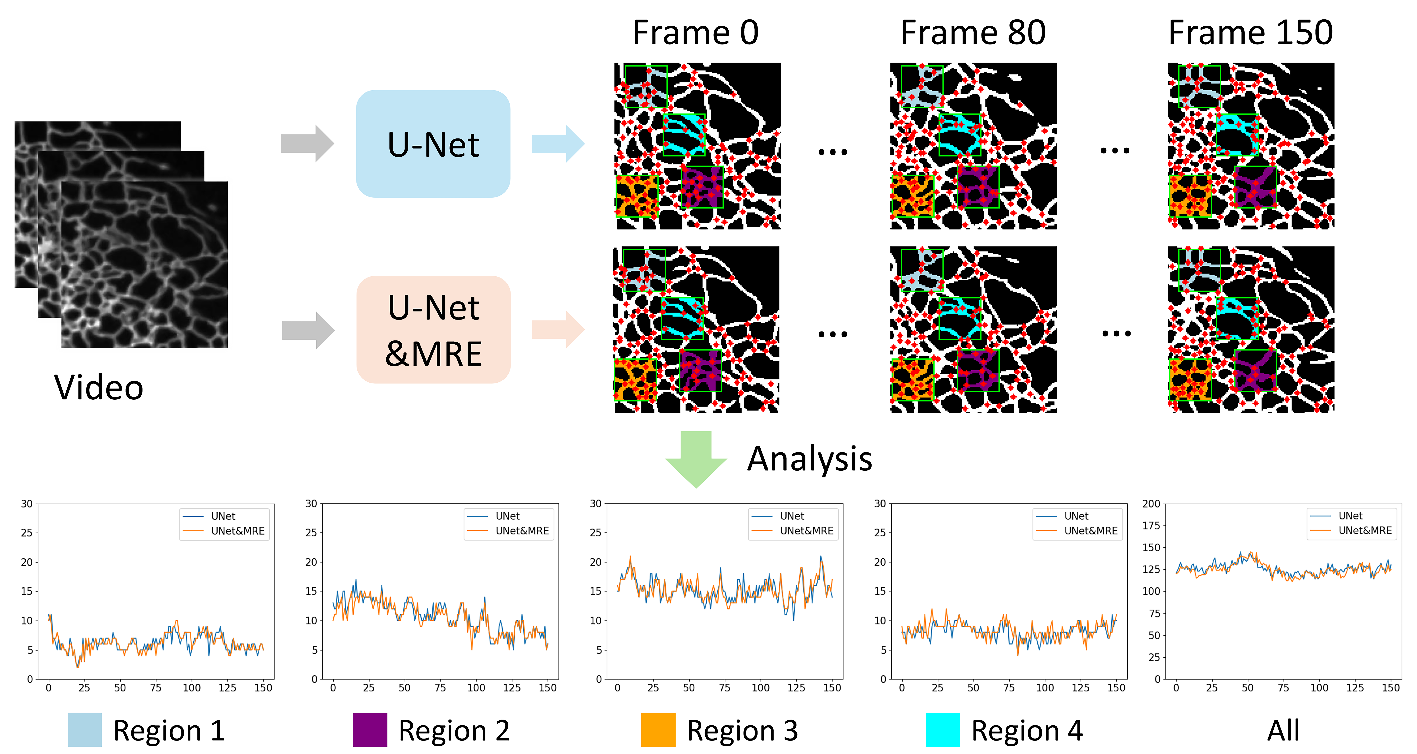


**Figure S3.** Characterization of consistency of segmentation results for the ER network using numbers of junctions. Segmentation of a live cell video from frame 0 to frame 150 was performed using U-Net and U-Net&MRE, respectively. Skeletonization was performed on the segmentation results of the ER network to identify and count junctions. The number of junctions was counted across consecutive frames in each selected region and line plots were generated. For each region, the X-axis label of the line plot indicates the frame index, the Y-axis label indicates the number of junctions.

**Characterizing model consistency in video segmentation:** To assess the consistency of the segmentation results across video frames, we selected four different $32\times32$ regions within the video frames and counted the numbers of junctions of the endoplasmic reticulum (ER) network in these regions. For the junction number within each region or the entire image, we calculated the standard deviation of its first-order difference (STD1), the standard deviation of its second-order difference (STD2), and its total variation (TV). Assuming a steady turnover of the ER network, we use these metrics to characterize the smoothness of the numbers of detected junctions. The results are summarized in Figure S3 and Table S4. Definitions of these metrics are given in the following.

Let $j_{i}$ denote the junction number in frame $i$, where$i\in[0, N]$ and *N* denotes the index of the last frame. It equals 150 in this case. The first-order difference between consecutive frames is calculated as:

$$\Delta j_{i}= j_{i+1}- j_{i}, i=0,1,\ldots,N-1$$

Subsequently, the second-order difference is calculated based on the results obtained from the first-order differences:

$$\Delta\left( \Delta j_{i} \right)= \Delta j_{i+1}- \Delta j_{i}, i=0,1,\ldots,N-2$$

Finally, we calculated the standard deviation of both the first-order and the second-order differences to evaluate the consistency of the number of junctions across the frames.

Total variation is another metric that is used widely in signal and image processing to quantify the smoothness of a signal or an image. We calculated the total variation of the junction counts using the following formula:

$$TV=\sum_{i=0}^{N-1} \left| j_{i+1}- j_{i} \right|,$$

Based on the segmentation results shown in Figure S3 and Table S4, we can conclude that by leveraging topological similarities of the structures, U-Net&MRE achieves more consistent segmentation than U-Net across video frames. Specifically, U-Net&MRE model shows lower STD1, STD2, and TV metrics in Region 1, Region 2, Region 3, and the entire image than U-Net. Furthermore, we conducted a two-sample two-sided student’s t-test on the number of junctions of two models. The p-value from the t-test on the junction counts under U-Net&MRE versus U-Net in the entire image was 9.12e-06 ($p<0.001$). This indicates that the difference between the segmentation results of two models is statistically significant. Overall, integration of MRE with U-Net increases the consistency of segmentation across video frames.

Table S4. Analysis of the consistency of video frame segmentation results. Based on the junction count of each region and entire image, the standard deviation of first-order difference (STD1), the standard deviation of second-order difference (STD2), and the total variation (TV) of the junction counts were calculated. Bold numbers indicate smaller values and better consistency.

| Model | Region1 | | | Region2 | | | Region3 | | | Region4 | | | All | | |
| --- | --- | --- | --- | --- | --- | --- | --- | --- | --- | --- | --- | --- | --- | --- | --- |
|  | STD1 | STD2 | TV | STD1 | STD2 | TV | STD1 | STD2 | TV | STD1 | STD2 | TV | STD1 | STD2 | TV |
| U-Net | 1.38 | 2.35 | 150 | 1.58 | 2.70 | 180 | 1.79 | 2.85 | 211 | **1.24** | **2.06** | **136** | 4.98 | 8.39 | 591 |
| U-Net&MRE | **1.13** | **1.92** | **121** | **1.57** | **2.64** | **177** | **1.55** | **2.38** | **179** | 1.45 | 2.42 | 164 | **4.23** | **6.79** | **491** |

1. Benchmarking latency introduced by MRE and $\mathbf{L}_{\mathbf{h}\mathbf{f}}$ on of inference time.

We measured the total inference time and per-frame inference time of U-Net and U-Net&MRE during video segmentation on an NVIDIA GeForce RTX 3090 GPU. The results are summarized in Table S5. Overall, the additional latency introduced by the MRE and $\mathbf{L}_{\mathbf{h}\mathbf{f}}$ is small, at about 1.4 millisecond per frame of 128×128 pixels.

Table S5. Inference time of U-Net and U-Net&MRE for video segmentation. Note: The size of each frame is 128×128. The total number of frames is 151.


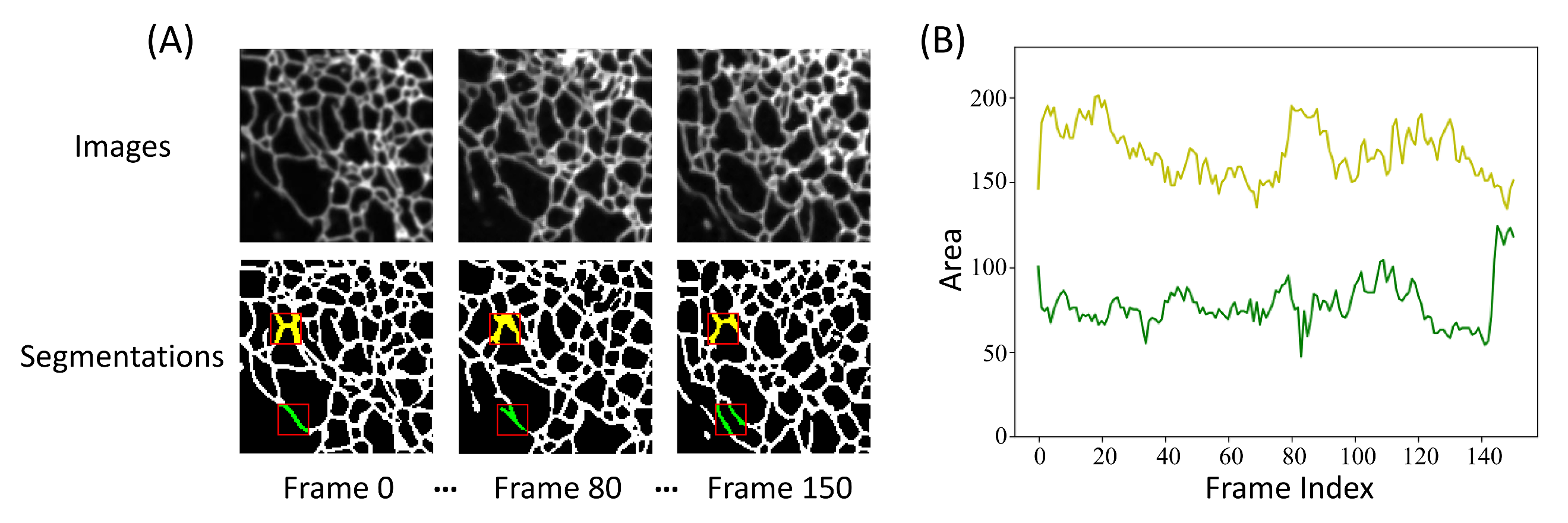
**Figure S4.** Morphology Analysis of ER network. (A) Images of an ER network section and corresponding segmentation results in different frames. (B) Areas of the network regions shown in yellow and green, respectively, over time within the two square boxes in (A).

| Model | Total Time (s) | Per-frame (s) |
| --- | --- | --- |
| U-Net | 0.506851 | 0.003357 |
| U-Net&MRE | 0.714943 | 0.004735 |

1. Morphological analysis of the ER network.

Accurate segmentation of the ER network enables quantitative analysis of its dynamic morphological properties in live cells. Figure S1A shows an ER network section in a live COS-7 cell and corresponding segmentation results in selected frames. Figure S1B shows calculated areas of the network regions shown in green and yellow, respectively, within the two square boxes in Fig. S1A.

1. Supplementary Video 01.

The video compares the segmentation result and downstream network analysis of our model versus the U-Net model.
